# Circulating miR-497 and miR-663b in plasma are potential novel biomarkers for bladder cancer

**DOI:** 10.1038/srep10437

**Published:** 2015-05-27

**Authors:** Mulong Du, Danni Shi, Lin Yuan, Pengchao Li, Haiyan Chu, Chao Qin, Changjun Yin, Zhengdong Zhang, Meilin Wang

**Affiliations:** 1 Department of Environmental Genomics, Jiangsu Key Laboratory of Cancer Biomarkers, Prevention and Treatment, Cancer Center, Nanjing Medical University, Nanjing, China; 2 State Key Laboratory of Reproductive Medicine, Institute of Toxicology, Nanjing Medical University, Nanjing, China; 3 Department of Urology, Jiangsu Province Hospital of TCM, Nanjing, China; 4 Department of Urology, the First Affiliated Hospita of Nanjing Medical University, Nanjing, China; 5 Department of Genetic Toxicology, the Key Laboratory of Modern Toxicology of Ministry of Education, School of Public Health, Nanjing Medical University, Nanjing, China

## Abstract

MicroRNAs (miRNAs), abundant and highly stable in the plasma, have been widely reported. This greatly pursued us to investigate whether plasma miRNAs could be considered as powerful biomarkers for diagnosing bladder cancer (BC). We performed a plasma miRNAs profile with the TaqMan Low Density Array, and a two-phase validation to detect the candidate miRNAs expression by quantitative PCR. The receiver operating characteristic curve (ROC) and the area under curve (AUC) were used to evaluate diagnostic accuracy. A total of eight plasma miRNAs abnormally expressed between BC patients and healthy controls in microarray analysis (i.e., elevated miRNAs for miR-505, miR-363 and miR-663b, and decreased for miR-99a, miR-194, miR-100, miR-497 and miR-1 in BC plasma). In further independent cohorts, miR-497 and miR-663b with significantly differential expression were confirmed. Moreover, the AUC, sensitivity and specificity were raised to 0.711 (95% CI = 0.641-0.780), 69.7% and 69.6%, respectively, when miR-497 and miR-663b were integrated. This is the first study systematically exploring the existence of specific plasma miRNAs as early diagnostic biomarkers for BC in Chinese population; and these findings supported that plasma miR-497 and miR-663b could be promising novel circulating biomarkers in clinical detection of BC.

Bladder cancer (BC) is one of the most common urinary system malignancies worldwide^1^. The ratio of male to female for BC patients is approximately 3:1, and the incidence and mortality of BC have obviously increased in recent years^2^. Although many studies have demonstrated several key molecules and pathways involved in the BC occurrence and development^3^,^4^, little is known about the exact mechanisms of BC etiology. Recently, extensive biomarkers, whatever from urine or peripheral blood, have been investigated for BC diagnosis, but their sensitivity and specificity is still not comparable with cystoscopy^5^,^6^,^7^. However, cystoscopy, even as gold standard, is invasive and costly, and its feelings of unpleasantness limit its wide application. Therefore, there is great need to find new noninvasive biomarkers to improve the early detection of BC.

MicroRNAs (miRNAs), a class of small non-coding RNAs, are involved in regulating a variety of biologic processes, including cell proliferation, differentiation and apoptosis^8^,^9^. Accumulating evidences have revealed that deregulation of miRNAs contribute to several kinds of disease, especially cancer development and progression^10^,^11^,^12^. Recently, some studies have investigated the alteration of some specific miRNAs in bladder carcinogenesis, indicating that deregulated miRNAs, modulating the expression of oncogenes/tumor suppressor genes, were involved in the occurrence and progression of BC, as well as prognosis^13^,^14^. Intriguingly, several miRNAs, previously identified in cells and tissues, were easily detected in extracellular fluids (e.g. plasma, serum or seminal plasma)^15^,^16^; especially, the plasma miRNAs have widely drawn the researchers’ attention for their unique merits: detectability, stability and tumor specificity^17^,^18^,^19^. To date, it has been identified several differentially expressed plasma miRNAs inferring to BC diagnosis^20^,^21^, but no studies were performed in Chinese BC patients. Thus, we perform a systematic study to explore whether some specific plasma miRNAs could be served as early diagnostic biomarkers for BC in China.

In present study, we systematically screened plasma miRNAs expression by using the TaqMan Low Density Array (TLDA) chips, and validated the candidate miRNAs in two further independent phases. According to receiver operating characteristic curve analysis and functional studies, we aimed to find a promising circulating biomarker in BC diagnosis.

## Results

### Patient Characteristics

The characteristics of enrolled participants are presented in Table 1. There were no differences between patients and controls on age, sex, smoking status, pack-years of smoking and drinking status (all *P* > 0.05). For the two-phase validation subjects, the majority of patients were in G1 (42.9% and 40.4%, respectively) and a large proportion of patients suffered from superficial bladder cancer (75% and 65.1%, respectively).

### Determination of endogenous controls for quantification of plasma miRNAs

To select a reliable endogenous control for quantification of plasma miRNA, we examined miR-16 and RNU6B levels in 48 plasmas, which were compared with spiked-in cel-miR-39. There were no significant difference in the levels of miR-16 (*P* = 0.575) and RNU6B (*P* = 0.284) between BC plasma and healthy plasma (data not shown). Then, we detected the stability of both miR-16 and RNU6B in plasma, showing that miR-16 expression level did not change obviously along with prolonged incubation time at room temperature with which the C_T_ value was around 20; while, RNU6B appeared to be less stable in plasma, of which the expression levels declined after prolonged incubation (*P* < 0.05) and the C_T_ value was over 35 (Fig. S1a). Therefore, we selected miR-16 as the endogenous control in the further analysis for its higher stability and abundance.

### Identification of differential expressed plasma miRNAs in discovery phase

In the discovery phase, a strategy for effective identification plasma miRNAs was conducted by using qRT-PCR-based miRNAs expression profiling arrays. According to the assessment, seven differential expressed plasma miRNAs were identified for next analysis: three elevated miRNAs (miR-663b, miR-363 and miR-505) and four decreased miRNAs (miR-99a, miR-194, miR-497 and miR-100) in BC plasma (Fig. S1b). We included miR-1 in the further analysis for its reported association with bladder cancer, although it did not meet the criteria (|∆∆C_T_| = 4.447) (Table S1). Finally, eight differentially expressed miRNAs were identified as candidates for further validation via qRT-PCR. Additionally, the cluster analyses notably revealed these eight significantly differentially expressed miRNAs (Fig. S2).

### Determination of candidate miRNAs in validation phases

**Figure 1:**
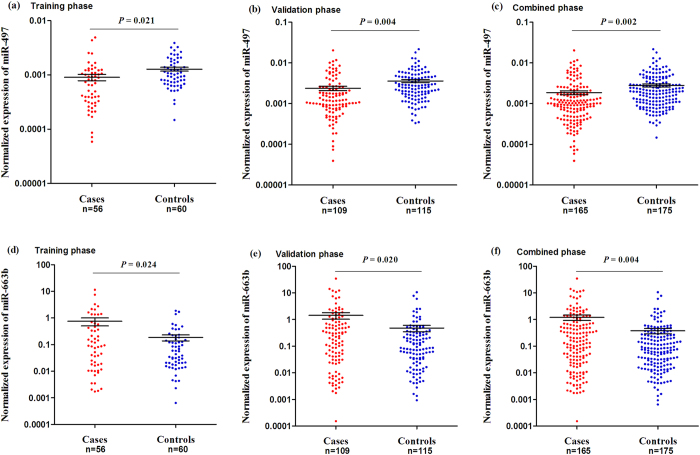
Two independent validations of plasma miR-497 and miR-663b. (**a**) and (**d**) for the discovery phase, (**b**) and (**e**) for validation phase, (**c**) and (**f**) for merged groups. The plasma miRNAs expressions were normalized to miR-16. The line represents the means value.

Totally, we performed a two-phase validation to examine the BC diagnostic accuracy of plasma miRNAs. In training phase, up to 56 BC plasma and 60 healthy plasma were enrolled to detect the candidate miRNAs, of which the expression levels were normalized to endogenous miR-16, showing that only two miRNAs (miR-497 and miR-663b) existed significantly differential expression between cases and controls (*P* < 0.05; Fig. 1a,d). Subsequently, a total of 109 patients and 115 controls were enrolled in the next validation phase. In line with the previous findings, miR-497 and miR-663b differentially expressed between cases and controls (Fig. 1b,e); and their exotic expressions were more significant when two validations were integrated (Fig. 1c,f).

**Figure 2:**
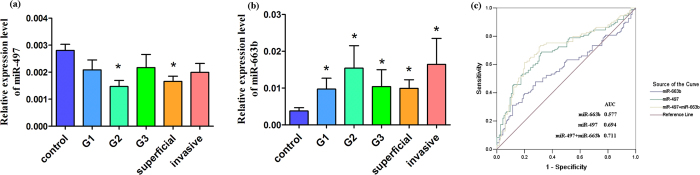
Expression of plasma miR-497 and miR-663b in clinical variables and ROC curves analysis. (**a**) for plasma miR-497 expression in clinical features and (**b**) for miR-663b; (**c**) for ROC curves analysis of each miRNA signature. Tumor grade included G1, G2 and G3; and tumor stage was divided into superficial (pT_a_-pT_1_) and invasive (pT_2_-pT_4_) stage. Statistically significant differences were determined using the Mann-Whitney U test. *P < 0.05 meant the plasma miRNAs were compared to the healthy controls’. The area under receiver operating characteristics (ROC) curve (AUC) value was calculated for distinguishing BC patients from normal control subjects.

In addition, we performed subgroup analysis by clinicopathologic features (tumor grade and stage), observing unexpectedly gradual variations in both plasma miR-497 and miR-663b (Fig. 2a,b and Table S3). Even though, the BC patients in G2 and superficial stage had decreased expression of miR-497 compared to the healthy controls (both *P* < 0.05, Fig. 2a); and significantly increased expression of miR-663b in each grade or stage (all *P* < 0.05, Fig. 2b).

Furthermore, we evaluated the sensitivity and specificity of each miRNA signature by generating ROC curves and calculating the area under ROC curve (AUC). As shown in Fig. 2c, the AUC of 0.694 (95% CI = 0.624-0.764) was in miR-497 and 0.577 (95% CI = 0.501-0.653) in miR-663b, respectively; nevertheless, the AUC was raised to 0.711 (95% CI = 0.641-0.780), and the sensitivity and specificity were increased to 69.7% and 69.6%, respectively, when miR-497 and miR-663b were assessed in combination. All these findings revealed that both plasma miR-497 and miR-663b might serve as useful biomarkers for distinguishing BC patients from healthy controls, especially their combination effect.

### Candidate miRNAs expression signature in BC tissues

**Figure 3:**
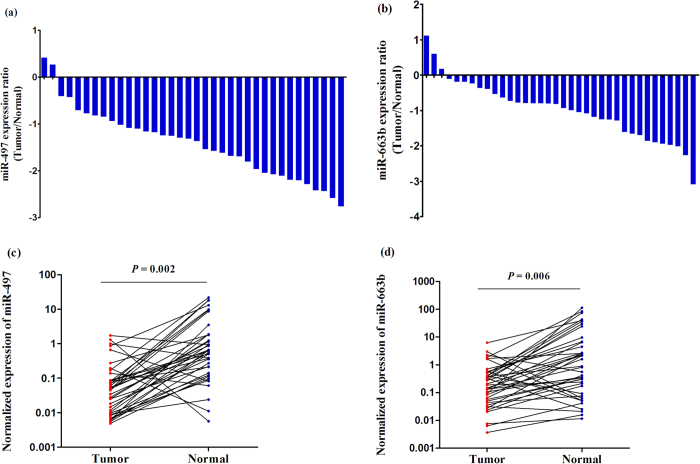
The miRNAs expressions in 36 BC tissues and adjacent non-malignant tissue samples. Logarithm of the ratio of (**a**) miR-497 and (**b**) miR-663b expression levels between tumor and normal tissues. Scatter plots of tissue levels of (**c**) miR-497 and (**d**) miR-663b in BC and its adjacent normal tissues, analyzed by the Wilcoxon signed rank test.

In order to further verify whether miR-497 and miR-663b expression in plasma were consistent with those in tissues, we detected these two miRNAs in 36 BC tissues and adjacent non-cancer tissues. The results showed that expression of miR-497 dramatically decreased in tumor tissues compared to non-malignant tissues, in accordance with that in plasma (Fig. 3a). However, surprisingly, miR-663b expression was unexpectedly significantly decreased in tumor tissues, opposite to results in plasma (Fig. 3b). Moreover, when compared these two miRNAs with clinicalpathological characteristics (Table S3), no significant correlation was identified between each miRNA and tumor grade/stage (all *P* > 0.05, Fig. S3). This might be for the small samples included; further validation with large samples was warranted.

### Effects of these two miRNAs on bladder cancer cellular phenotypes

To investigate whether these two miRNAs could affect phenotypes of BC cell line, we performed a gain-of-function study by transient transfection of miRNAs mimics into EJ cell line. For miR-497, more apoptotic cells were identified in miR-497 mimic-transfected EJ cell compared to negative controls (34.28% of early apoptosis and 35.02% of late apoptosis in miR-497 vs. 16.15% of early apoptosis and 8.96% of late apoptosis in negative controls; Fig. S4a); while no significant differentials in cell cycle (Fig. S4b). For miR-663b, the EJ cell proliferation ability was accelerated; more migration and invasive cells and less apoptotic cells were observed when compared to negative controls (Fig. S4a/c/d); but not affect cell cycle (Fig. S4b).

## Discussion

In the present study, by screening the whole-genome plasma miRNAs between BC patients and cancer-free controls through TLDA technology, we identified eight differentially expressed plasma miRNAs. In the subsequent two-stage validation, we identified that miR-497 dramatically decreased in BC plasma, while miR-663b increased. Meanwhile, the results were also verified in the evaluation of the diagnostic performance of miRNAs in different BC stage and grade. Furthermore, ROC analysis was performed to assess the diagnostic accuracy, showing that plasma miR-497 and miR-663b presented a relative high sensitivity and specificity. All these suggested that miR-497 and miR-663b could be a promising class of noninvasive biomarkers for BC diagnosis. To our knowledge, this is the first study to comprehensively investigate the use of plasma miRNAs as a noninvasive measure of BC diagnosis in Chinese population.

Up to date, emerging evidences have demonstrated that several tumor specific features can be detected in human serum or plasma samples and be widely used in diagnosing serious diseases (e.g. alpha-fetoprotein for liver cancer^22^, prostate-specific antigen for prostate cancer^23^). However, there are still not specific diagnostic biomarkers for BC. Along with the developing of high-throughput genetic sequencing technology, numerous studies have explored miRNA expression profiles for BC, either in solid tissues or in body fluids. Lourdes *et al.* conducted a miRNAs expression profile from urines of bladder urothelial cell carcinoma (UCC) patients, and identified that a sort of six miRNAs (i.e., miR-187, miR-18a*, miR-25, miR-142-3p, miR-140-5p, and miR-204) could correctly classify UCC patients. Even though it was an ideal non-invasive way for BC diagnosis, many researchers considered that urinary miRNAs were not specific to BC for its mixed composition of both filtered miRNAs (derived from the blood) and multiple cell types miRNAs (derived from lymphocytes, red blood cells, normal urothelial cells or tumor cells, etc.)^24^. Gildea *et al.* strongly indicated that plasma exosomal miRNAs could not go through the glomerulus excreting into the urine in appreciable quantities on account of their large size^25^. In that case, application of serum/plasma miRNAs seems more reliable in BC diagnosis.

Recent studies have showed that human serum/plasma could supply abundant novel promising noninvasive biomarkers for BC diagnosis. In 2012, Scheffer *et al.* selected 22 upregulated miRNAs in BC tissues to measure their levels in serum, and indicated that no serum miRNAs could be helpful in BC diagnosis, even though serum miR-141 and miR-639 levels were slightly increased in BC patients^21^. Furthermore, Adam *et al.* performed plasma miRNAs array analysis and observed some diagnostically relevant miRNAs for BC detection. These findings provided sufficient evidence supporting the use of circulating miRNAs for BC diagnosis; we are looking forward to the discovery of suitable circulating miRNAs for diagnosing BC.

It has been known that normalization is a critical step for the precise quantification of miRNAs levels, especially in circulation; and the proper normalization will eliminate systematic bias and experimental variation to ensure accurate detection of biological differences among samples. However, an urgent problem confusing researchers is how to confirm consensus endogenous controls. Hu *et al.* proposed the combination of miR-484 and miR-191 as potential endogenous control for serum miRNA detection^26^. In this study, we focused on two common endogenous controls: RNU6B and miR-16. Although no differential expression was found in plasma RNU6B and miR-16 between cases and controls, miR-16 was more stable than RNU6B at room temperature and thus more suitable for quantification miRNA levels as endogenous controls. Similarly, miR-16, confirmed as a stable endogenous control in plasma/serum, has been widely used in multiple tumors, including breast cancer^27^, ovarian cancer^28^ and colorectal cancer^29^.

In our study, miR-497 and miR-663b were found to have significantly abnormal expression in BC patients plasma. Several studies have reported that miR-497 as tumor suppressor could be a potential diagnostic marker for cancers^30^,^31^,^32^. Similar to our findings, Yang *et al.* demonstrated that seven identified miRNAs including miR-497 exhibited a global decrease in tumor tissues compared to normal tissues, and these serum miRNAs were further markedly increased after operation for malignant astrocytomas^31^. By using the bioinformatics methods, including TargetScan, miRanda and miRDB (Table S4), five genes were reported to be related to the cancer development and progression, such as significantly upregulated FGF2 levels in BC tissues compared to the normal^33^. Previous study has reported that increased miR-497 expression in BC cell lines could inhibit cell proliferation, migration and invasion^34^. Similarly, we observed that evaluated miR-497 level could facilitate BC cell apoptosis, but not cell cycle (Fig. S4a/b); all above findings supported our identification that decreased miR-497 expression in both BC plasma and tissue samples could exert as a suppressor gene in BC etiology.

However, as to miR-663b, its expression pattern and functional role in tumors has been controversial: downregulated in breast cancer^35^, glioblastoma^36^ and gastric cancer^37^; while upregulated in nasopharyngeal cancer^38^ and cutaneous T-cell lymphoma^39^. In this study, plasma miR-663b expression level was obviously elevated in BC patients, while the tumor tissues level was notably decreased. These interesting phenomena dramatically drew our attention for further functional study. We transfected miR-663b mimics into BC cell line and found that miR-663b could inhibit cell apoptosis; and promote cancer cell proliferation, migration and invasion; but not affect cell cycle (Fig. S4). These findings indicated that miR-663b might act role as an oncogene in BC cell line, in spite of its downregulated expression in BC tissues. This inverse relation seems to be the consequence of a dynamic regulation of the biology of miR-663b in the bladder. These interesting findings reflect a phenomenon of physiological significance in the biology of diseases, including BC and non-alcoholic fatty liver disease^40^.

This is the first study to screen plasma miRNAs profiles from BC patients in Chinese population. Major strengths of our study included the design of multi-stage validation, determination of endogenous controls for quantification of plasma miRNAs and rigorous replication using different systems (plasma and tissues). Nevertheless, some limitations should be concerned. Firstly, the sample size in validation phases was relatively small, which may present underpowered results; fortunately, the two-stage validation results were remarkably consistent. Secondly, the yielded AUC of 0.711, sensitivity of 69.7% and specificity of 69.6% by combination of plasma miR-497 and miR-663b did not achieve the best diagnostic accuracy, although they proved to be a suitable discrimination tool to a certain extent. Thirdly, to verify whether these miRNAs are special for BC, their expression levels should be compared and analyzed in other different kinds of cancers in the further study. Accordingly, we cannot claim completeness for miRNA profiles among BC patients and further large samples in multi-centers are needed to assess the potential of the reported miRNAs before clinical application.

In conclusion, our findings provided the first insight into the plasma miRNAs profiles in BC patients of Chinese and plasma miR-497 and miR-663b were supposed to be promising novel circulating biomarkers in clinical detection of BC. However, the clinical value and wide application of these two miRNAs in diagnosing BC still require further investigation and optimization.

## Material and Methods

### Study design and subjects

This study was approved by the institutional review board of Nanjing Medical University, and all subjects signed a written informed consent form. All experiments were performed in accordance with relevant guidelines and regulations.

A total of 175 plasma samples from BC patients and 185 from healthy controls were randomly selected from an ongoing study, which recruited subjects from the First Affiliated Hospital of Nanjing Medical University and Jiangsu Province Hospital of Traditional Chinese Medicine between January 2003 and October 2013. All cases were histologically confirmed suffering from bladder transitional cell carcinoma, and the healthy controls, frequency matched to cases on age and sex, were recruited from those who were seeking for health care in the same hospitals, and who were genetically unrelated cancer-free individuals. In present study, we designed a four-phase study (Fig. S5). Briefly, in the discovery phase, 10 BC plasma and 10 healthy plasma were randomly selected to perform the chips of TaqMan Low Density Array (TLDA). As further independent phases detected by TaqMan qPCR, a total of 56 cases and 60 controls were enrolled in training phase, and 109 cases and 115 controls in validation phase.

### Preparation of plasma and tissues and RNA extraction

Up to 5 ml whole blood were collected from each subject using an anticoagulant drying tube with Ethylene Diamine Tetraacetic Acid (EDTA). The collected blood were immediately separated into plasma and cellular fractions by centrifugation at 3,000 rpm for 5 min at 4 °C, followed by second centrifugation at 12,000 rpm for 15 min at 4 °C to completely remove cell debris. Then, the plasma was carefully stored into 1.5 ml non-RNAase centrifugal tube at −80 °C. The BC and its corresponding normal tissues were obtained from surgically removed specimens, and then immediately frozen in liquid nitrogen.

The RNA was isolated from 100 μl plasma by using the Trizol Reagent (Invitrogen, Carlsbad, CA, USA) for denaturizing and Qiagen miRNeasy Mini kit (Qiagen, Valencia, CA, USA) for RNA collection and purification following the manufacturer’s protocol. For normalization of sample-to-sample variation in RNA isolation efficiency, synthetic ath-miR-159a was added to each denatured sample for TLDA chips, and synthetic C. elegans miRNA-39 (cel-miR-39) with final concentration of 10^−4^  pmol/μl was added in each denatured sample for qRT-PCR analysis. The tissue RNAs were extracted by using mirVana miRNA Isolation Kit (Applied Biosystems, Foster City, CA, USA) according to the manufacturer’s protocol.

### TLDA chip assays and qRT-PCR

In discovery phase, plasma miRNA expression profiles between BC patients and healthy controls were performed using TLDA chips with 754 human miRNAs probes (human microRNA panel V2.0, Applied Biosystems, CA, USA), in which two pooled plasma samples with each 1ml (1ml = 100 μl/sample * 10 samples) was used. A pre-amplification step was conducted by using the TaqMan PreAmp Mastermix with default PCR procedure on an ABI 7900HT (Applied Biosystems, Foster City, CA, USA), and analyzed by using RQ manager 1.2 software (Applied Biosystems, Foster City, CA, USA). The relative miRNAs expression levels were normalized to ath-miR-159a. According to the scientific and applicable considerations, each miRNA at most 35 of C_T_ value and at least 5 of absolute value of ∆∆C_T_ (C_Tcase_ - C_Tcontrol_) was selected for further analysis^41^.

In further independent phases, total plasma RNA spiked in cel-miR-39 were reverse-transcribed to complementary DNA by using TaqMan MicroRNA Reverse Transcription Kit with stem-loop RT primers (Applied Biosystems, Foster City, CA, USA), and the quantification was performed using the TaqMan PCR kit. For tissue miRNAs, the quantification was based on SYBR Green method (TaKaRa Bio, Japan), and normalized to the expression levels of RNU6B. The primers used for amplification of miR-497 and miR-663b are shown in supplementary Table S2. All the quantification were on ABI 7900HT (Applied Biosystems, Foster City, CA, USA), and the reactions were incubated in a 384-well optical plate at 95 °C for 5 min, followed by 40 cycles of 95 °C for 15 s and 60 °C for 1 min. All reactions were run in triplicate.

### Endogenous controls for quantification of plasma miRNAs

To identify a reliable endogenous control for this study, we focused on two commonly used endogenous miRNAs (miR-16 and RNU6B). A total of 24 cases and 24 controls were selected to evaluate the differential expression of these two candidate endogenous plasma miRNAs. All these plasmas were processed under the exactly same conditions. To further assess the stability of these two endogenous miRNAs in plasma, we randomly selected 3 plasma samples and divided each of them into 4 parts, and further prolonged their incubation at room temperature for 0, 1, 2 and 4 h in nuclease-free tubes before RNA isolation, respectively. These two endogenous miRNAs were normalized to the spiked-in cel-miR-39.

### Functional study of miRNAs on bladder cancer cellular phenotypes

EJ cells were transfected with miRNAs mimics or negative controls by Lipofectamine 2000 (Invitrogen). For apoptosis analysis, cells were collected after 48 h transfection and stained using Annexin V-FITC Apoptosis Detection Kit (Invitrogen); and for cycle detection, celled were fixed in 75% alcohol after 48 h transfection, and stained with propidium iodide (Sigma). All above experiments were examined by FACS Calibur Flow Cytometry (BD Biasciences), and three independent experiments were conducted in triplicate. For proliferation analysis, the proliferation ability of EJ cells after transfections was assayed using Cell Counting Kit-8 (Dojindo) according to the manufacturer’s protocol. For cell migration and invasion assay, 6h-transfected cells were seeded in the upper chamber non-coated or coated with Matrigel (BD Biosciences) for migration or invasion, respectively, in 24-well dishes; and DMEM medium (Invitrogen) supplemented with 10% fetal bovine serum was placed in the lower chamber. After 48 h incubated, cells were fixed in 95% methyl alcohol and stained with crystal violet for 20 min at room temperature; and after the upper side cells were removed and washed, the cells on the lower side were as migration or invasion cells.

### Statistical analysis

Differences in demographic and clinical characteristics between cases and controls were evaluated by using the Student’s *t*-test (for continuous variables) or Pearson’s χ^2^-test (for categorical variables). The SDS 2.3 software (Applied Biosystems, Foster City, CA, USA) was used to calculate the threshold cycle (C_T_) value; non-parametric Mann-Whitney test was used for the unpaired two groups comparison, Wilcoxon signed rank test for paired two groups, and Kruskal-Wallis test for multiple groups. The receiver operating characteristic (ROC) curve was constructed to determine the sensitivity, specificity and area under curve (AUC) for plasma miRNA levels. A *P-*value of less than 0.05 was considered statistically significant, and all tests were two tailed. All the statistical analyses were done with Statistical Analysis System software (9.1.3; SAS Institute, Inc., Cary, NC, USA).

## Additional Information

**How to cite this article**: Du, M. *et al.* Circulating miR-497 and miR-663b in plasma are potential novel biomarkers for bladder cancer. *Sci. Rep.* **5**, 10437; doi: 10.1038/srep10437 (2015).

## Supplementary Material

Supplementary Information

## Figures and Tables

**Table 1:** Distribution of selected variables between the bladder cancer cases and healthy control individuals.

**Variables**	**Training phase**		***P***^a^	**Validation phase**		***P***^a^
	**cases (n = 56)N (%)**	**controls (n = 60)N (%)**		**cases (n = 109)N (%)**	**controls (n = 115)N (%)**	
Age (years) (mean±SD)	64.5 ± 11.9	67 ± 11.5	0.329	64.6 ± 11.7	62.3 ± 13.1	0.168
						
Sex						
Male	43 (76.8)	42 (70.0)	0.409	87 (79.8)	82 (71.3)	0.139
Female	13 (23.2)	18 (30.0)		22 (20.2)	33 (28.7)	
						
Smoking status						
Never	32 (57.1)	41 (68.3)	0.212	71 (65.1)	62 (53.9)	0.087
Ever	24 (42.9)	19 (31.7)		38 (34.9)	53 (46.1)	
						
Pack-years of smoking						
0	32 (57.2)	41 (68.3)	0.453	71 (65.1)	62 (53.9)	0.184
0-20	12 (21.4)	9 (15.0)		18 (16.5)	29(25.2)	
> 20	12 (21.4)	10 (16.7)		20 (18.4)	24 (20.9)	
						
Tumor grade						
G1	24 (42.9)			44 (40.4)		
G2	21 (37.5)			40 (36.7)		
G3	11 (19.6)			25 (22.9)		
						
Tumor stage						
Superficial (pT_a_-pT_1_)	42 (75.0)			71 (65.1)		
Invasive (pT_2_-pT_4_)	14 (25.0)			38 (34.9)		

^a^Student’s t-test for age distributions between cases and controls; Two-sided χ^2^ test for other selected variables between cases and controls. SD, standard deviation.
