# Supplementary material for: Circulating miR-497 and miR-663b in plasma are potential novel biomarkers for bladder cancer
Source: Sci Rep. 2015 May 27;5:10437. doi: 10.1038/srep10437 (PMC4444850; doi:10.1038/srep10437)
Supplement: Supplementary Information [file srep10437-s1.doc]

**Circulating miR-497 and miR-663b in plasma are potential novel biomarkers for bladder cancer**

Mulong Du^1,2†^, Danni Shi^1,2†^, Lin Yuan^3†^, Pengchao Li^4^, Haiyan Chu^1,2,5^, Chao Qin^4^, Changjun Yin^4^, Zhengdong Zhang^1,2,5*^ , Meilin Wang^1,2,5*^

Author’s Affiliations:

^1^Department of Environmental Genomics, Jiangsu Key Laboratory of Cancer Biomarkers, Prevention and Treatment, Cancer Center, Nanjing Medical University, Nanjing, China

^2^State Key Laboratory of Reproductive Medicine, Institute of Toxicology, Nanjing Medical University, Nanjing, China

^3^Department of Urology, Jiangsu Province Hospital of TCM, Nanjing, China

^4^Department of Urology, the First Affiliated Hospita of Nanjing Medical University, Nanjing, China

^5^Department of Genetic Toxicology, the Key Laboratory of Modern Toxicology of Ministry of Education, School of Public Health, Nanjing Medical University, Nanjing, China

^†^These authors contribute equally to this work.

***Correspondence to:** Zhengdong Zhang, Department of Environmental Genomics, School of Public Health, Nanjing Medical University, 818 East Tianyuan Road, Jiangning District, Nanjing 211166, China. Tel.: + 86 25 86868423; fax: + 86 25 86868499. E-mail address: drzdzhang@gmail.com and Meilin Wang, Department of Environmental Genomics, School of Public Health, Nanjing Medical University, 818 East Tianyuan Road, Jiangning District, Nanjing 211166, China. E-mail: mwang@njmu.edu.cn

**Short title:** Plasma miRNAs and bladder cancer

**Keywords**: Bladder cancer, Biomarker, MicroRNA, Plasma

**Table S1.** Selection of eight miRNAs in the discovery stage

| miRNAs | Cases (n=10) |  | Controls (n=10) | Fold change ^a^ |
| --- | --- | --- | --- | --- |
|  | ΔC_T_ |  | ΔC_T_ |  |
| **hsa-miR-663b** | 15.195 |  | 23.255 | 266.8712 |
| hsa-miR-363 | 10.607 |  | 17.657 | 132.5139 |
| hsa-miR-505 | 17.947 |  | 22.921 | 31.42847 |
| hsa-miR-99a | 16.548 |  | 9.806 | 0.009342 |
| hsa-miR-194 | 19.237 |  | 13.038 | 0.013612 |
| **hsa-miR-497** | 20.114 |  | 14.415 | 0.01925 |
| hsa-miR-100 | 16.168 |  | 10.914 | 0.026205 |
| hsa-miR-1 ^b^ | 20.911 |  | 16.464 | 0.045848 |

^a^ Fold change equals to 2^-∆∆CT^.

^b^ has-miR-1 was reported to be associated with bladder cancer, but it did not meet the criteria (|∆∆C_T_|＝4.447)

**Table S2.** Primer sequences of miRNAs

| miRNAs | Primer sequences（5’-3’） |
| --- | --- |
| has-miR-497 | CTCAACTGGTGTCGTGGAGTCGGCAATTCAGTTGAGACAAACCA |
| has-miR-663b | CTCAACTGGTGTCGTGGAGTCGGCAATTCAGTTGAGCCTCAGGC |
| RNU6B | AACGCTTCACGAATTTGCGT |

**Table S3.** The miRNAs expression level in each BC pathological parameters

| miRNAs | BC pathological parameters | | | | | | Healthy controls |
| --- | --- | --- | --- | --- | --- | --- | --- |
|  | Grades | | |  | Stages | |  |
|  | G1 | G2 | G3 |  | superficial | invasive |  |
| Plasma |  |  |  |  |  |  |  |
| miR-497 | 0.0021 ± 0.0004 | 0.0015 ± 0.0002 | 0.0022 ± 0.0005 |  | 0.0017 ± 0.0002 | 0.0020 ± 0.0003 | 0.0028 ± 0.0002 |
| miR-663b | 0.0097 ± 0.0029 | 0.0154 ± 0.0061 | 0.0104 ± 0.0046 |  | 0.0099 ± 0.0023 | 0.0164 ± 0.0071 | 0.0038 ± 0.0009 |
| Tissue |  |  |  |  |  |  |  |
| miR-497 | 0.0023 ± 0.0012 | 0.0007 ± 0.0003 | 0.0015 ± 0.0009 |  | 0.0019 ± 0.0009 | 0.0013 ± 0.0007 |  |
| miR-663b | 0.0071 ± 0.0038 | 0.0027 ± 0.0008 | 0.0065 ± 0.0025 |  | 0.0060 ± 0.0028 | 0.0055 ± 0.0020 |  |

All data here presented mean ± SEM.

**Table S4.** Bioinformatics methods predicting targets of miR-497

| Target genes | Full name | TargetScan  (context score) | Miranda  (mirSVR score) | MiRDB  (Target Score) | Related cancers |
| --- | --- | --- | --- | --- | --- |
| **CCNE1** | cyclin E1 | -0.72 | -2.01 | 93 | **bladder cancer**; breast cancer; ovarian cancer |
| **UBE2Q1** | ubiquitin-conjugating enzyme E2Q family member 1 | -0.74 | -0.71 | 74 | breast cancer; gastric cancer; colorectal cancer |
| **PISD** | phosphatidylserine decarboxylase | -0.73 | -0.69 | 71 | colorectal cancer |
| **FGF2** | fibroblast growth factor 2 (basic) | -1.54 | -1.40 | 98 | **bladder cancer**; ovarian cancer; non small cell lung cancer; gastric cancer; breast cancer; |
| **PLAG1** | pleiomorphic adenoma gene 1 | -0.85 | -1.75 | 89 | myoepithelial salivary gland tumour; pleomorphic adenoma |
| LC11A2 | Solute carrier family 11 (proton-coupled divalent metal ion transporters), member 2 | -1.07 | -1.04 | 81 |  |
| PLSCR4 | phospholipid scramblase 4 | -0.80 | -1.05 | 88 |  |
| ANO3 | anoctamin 3 | -0.80 | -1.06 | 95 |  |
| UNC80 | unc-80 homolog (C. elegans) | -0.78 | -1.16 | 78 |  |
| CD80 | CD80 molecule | -0.73 | -0.87 | 96 |  |

**Figure S1.** Stability of RNU6B and miR-16 in plasma by prolonged incubation at room temperature for 0 h, 1 h, 2 h, and 4 h (a); Expression levels of candidate eight plasma miRNAs in BC and healthy controls (b).

**Figure S2**. Heat map representation of the eight miRNAs model for bladder cancer in the 116 samples examined in the training phase.

**Figure S3.** Expression of tissue miR-497 and miR-663b in clinical features. (a) for tissue miR-497 expression in clinical characteristics and (b) for miR-663b.

**Figure S4.** Cellular phenotypes of EJ cell line by transfected with miRNAs mimics. (a) EJ cells transfected with miR-497 or miR-663b mimics were stained and examined by flow cytometry. The right upper quadrant represented late apoptotic cells percentage; and the right lower for apoptotic cells percentage. (b) Representative results of EJ cell cycle transfected with each miRNAs mimics, analyzed by flow cytometry. (c) Cell proliferation of EJ cell line transfected with miR-663b mimics was measured by the CCK8 assay. **, *P* < 0.01 in 48 hours transfection. (d) Increased level of miR-663 accelerated EJ cell migration and invasion.

**Figure S5**. Summary of this study design.

**Figure S1**

**
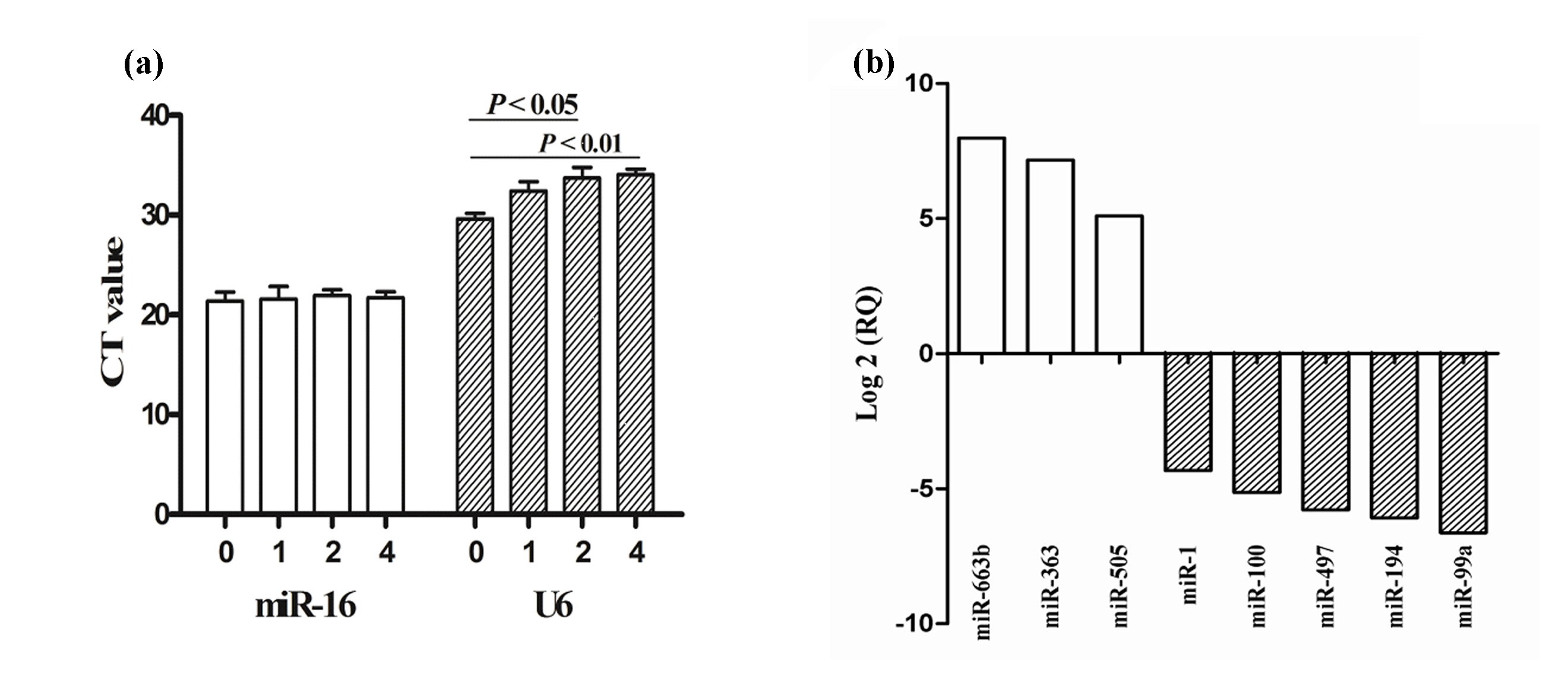
**

**Figure S2**

**
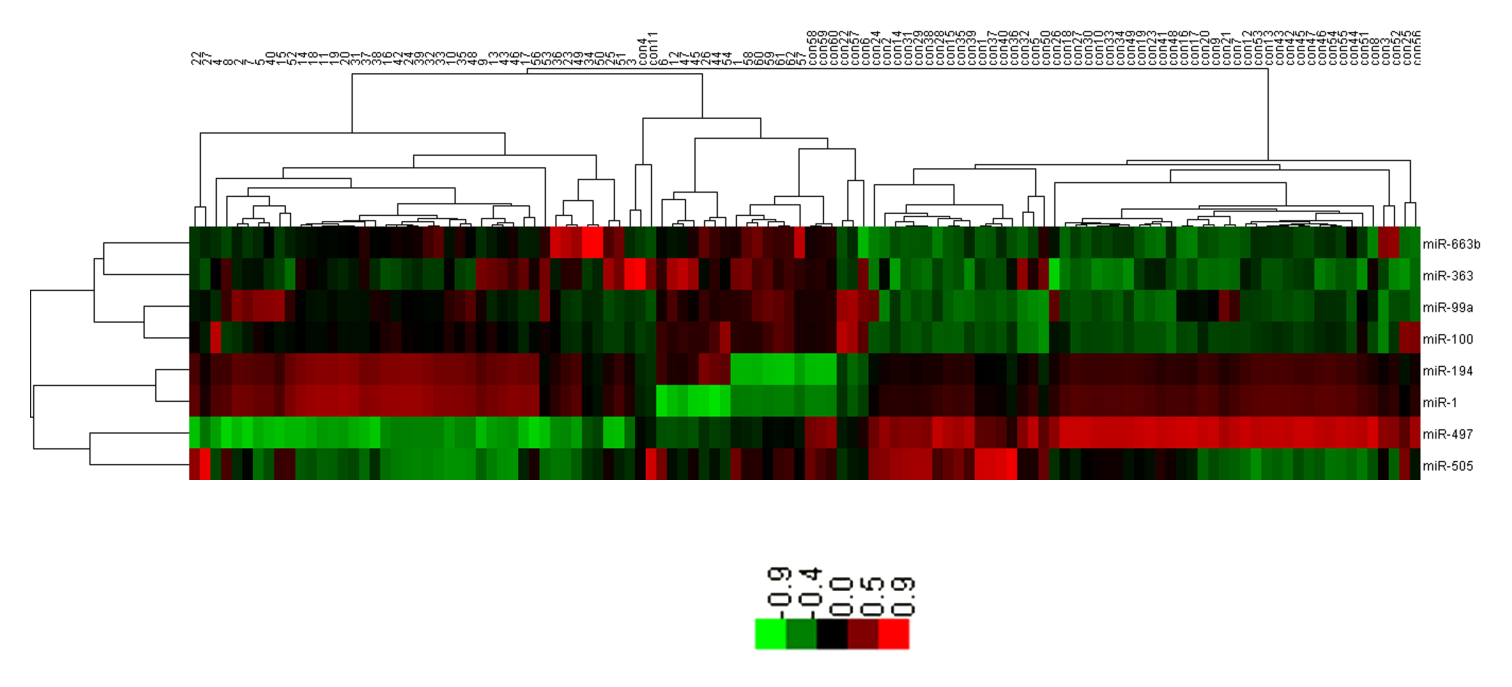
**

**Figure S3**

**
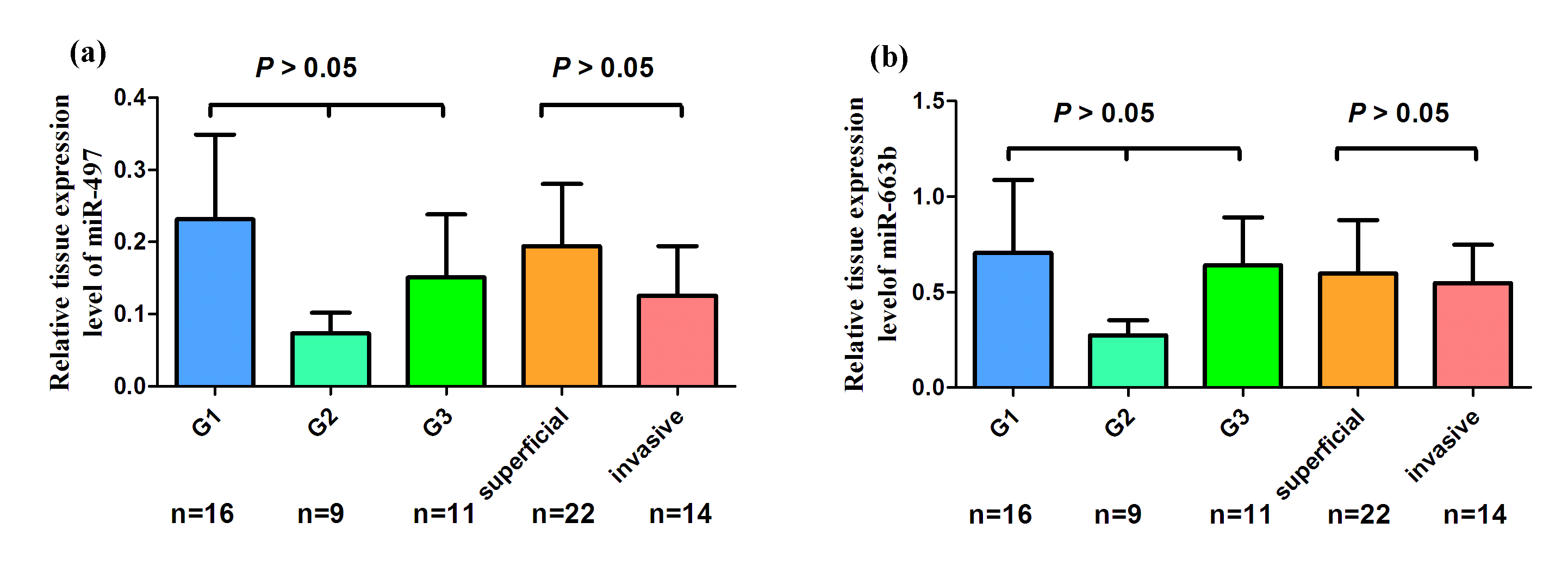
**

**Figure S4**

**
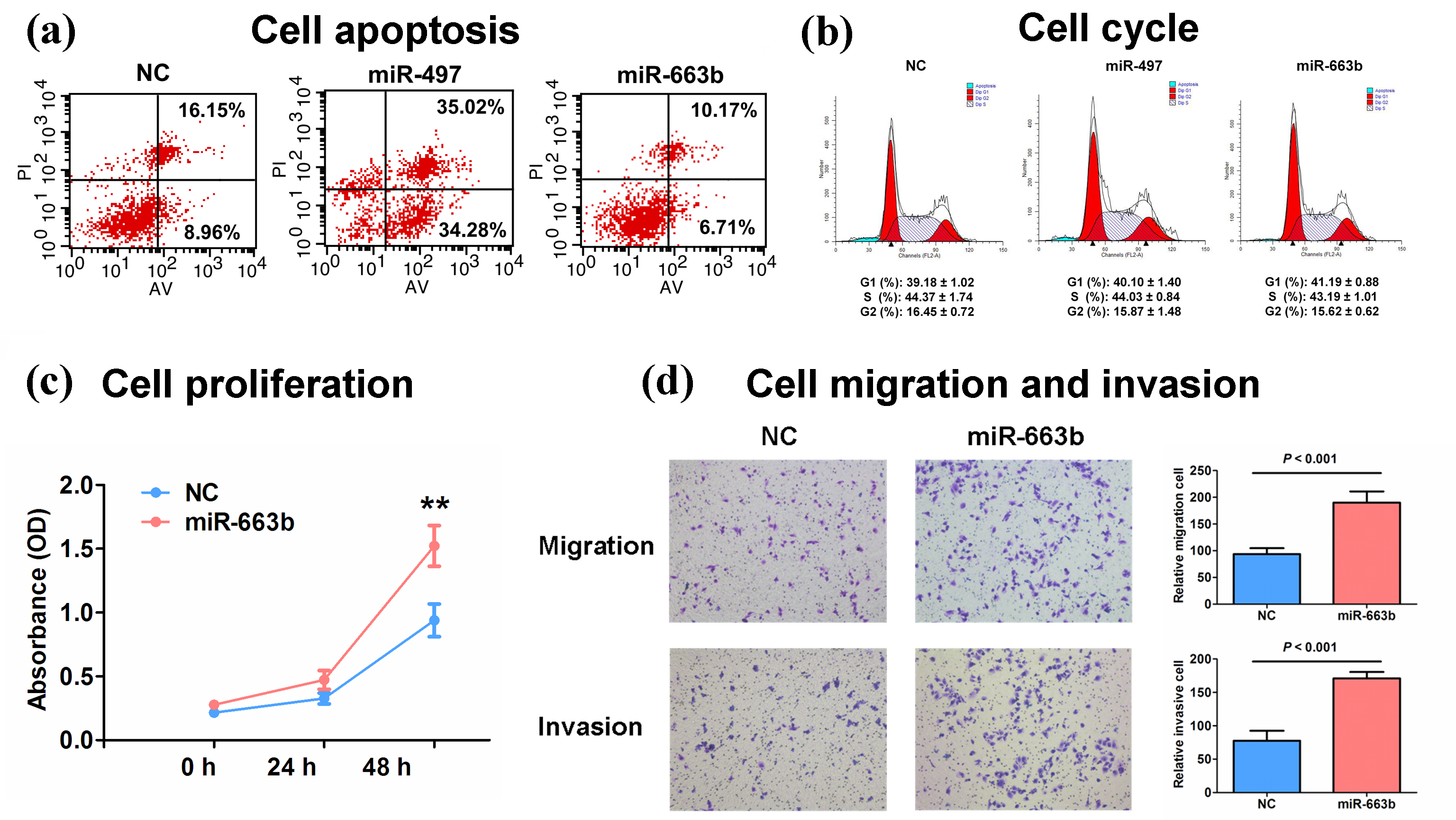
**

**Figure S5**

**
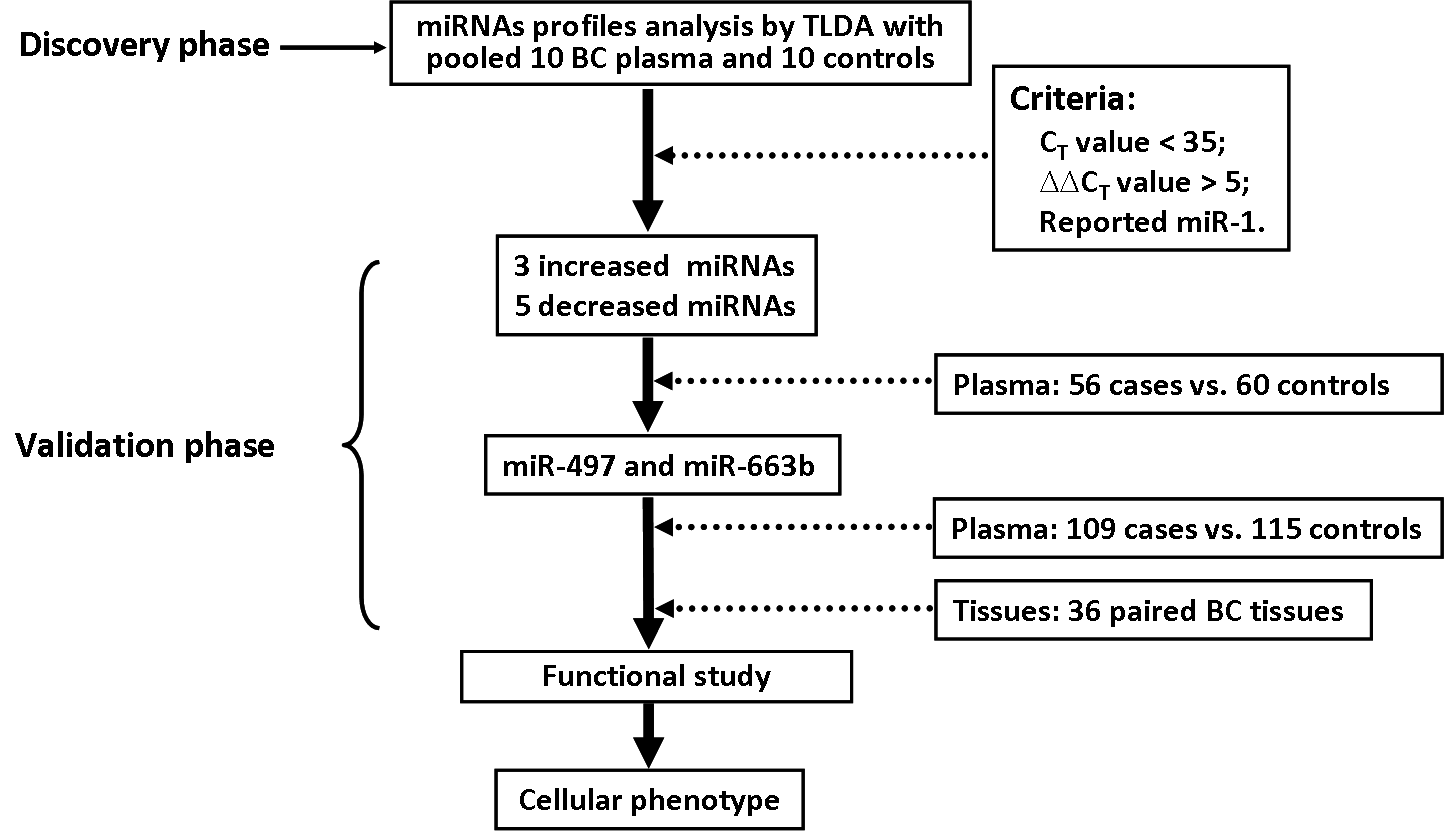
**
